# P02.54. Tools from the SPICER project for doing clinic-based research on integrative medicine and CAM

**DOI:** 10.1186/1472-6882-12-S1-P110

**Published:** 2012-06-12

**Authors:** M Aickin

**Affiliations:** 1 University of Arizona, Tucson, USA

## Purpose

Non-intervention research using clinical records is difficult, both from the theoretical and practical standpoints. Progress on the former requires progress on the latter. Several techniques have been developed in the SPICER project to overcome the various practical problems encountered in research from electronic medical records (EMRs). This presentation will exhibit techniques that have been developed and appear to work well.

## Methods

Non-intervention projects on integrative medicine and pain are underway, one at a large HMO and another in a mixed conventional/CAM clinic. Both of these projects have addressed problems of refining research objectives, performing data extraction, and designing analyses.

## Results

In-depth understanding of the EMR system, the clinic, and how practitioners use the system are necessary to identify patient samples and available information. A successful strategy for obtaining research datasets from EMR has been based on a radical simplification of the interface between the IT professionals and the analysis professionals. The post-extraction data manipulation burden is somewhat high, but must be faced because conventional data management techniques are infeasible. The well-known ICD9 and CPT codes can be simplified in medically appropriate ways, but EMRs often introduce non-standard features into these systems. Medications present unique problems, but the ACT (anatomic, therapeutic, chemical) annotation system is very useful in analysis datasets. Therapeutically relevant analyses must account for treatment selection bias. A reading list of resources will be provided.

## Conclusion

EMR-based research is potentially an extremely valuable complement to other methods of medical investigation. Developing this alternative source of therapeutic knowledge requires dissemination of a toolkit that solves the practical problems.

